# Lead removal from water – dependence on the form of carbon and surface functionalization†

**DOI:** 10.1039/c8ra02264j

**Published:** 2018-05-18

**Authors:** Chun Sing Kam, Tik Lun Leung, Fangzhou Liu, Aleksandra B. Djurišić, Mao Hai Xie, Wai-Kin Chan, Ying Zhou, Kaimin Shih

**Affiliations:** Department of Physics, The University of Hong Kong Pokfulam Hong Kong dalek@hku.hk; Department of Chemistry, The University of Hong Kong Pokfulam Hong Kong; Department of Civil Engineering, The University of Hong Kong Pokfulam Hong Kong

## Abstract

We have investigated lead adsorption on different forms of nanostructured carbon, namely multiwall carbon nanotubes (MWCNT) and reduced graphene oxide (RGO) functionalized with different functional groups (hydroxyl, carboxyl, and amino groups). We found that the same functional group does not result in the same performance trends for different nanostructured carbons. Drastically different behavior was observed for the amino-group functionalization, where a significant improvement is observed for MWCNT, while worse performance compared to non-functionalized material is obtained for RGO. On the other hand, hydroxyl and carboxyl group functionalization improves the lead adsorption regardless of the form of carbon. The best performing RGO sample, namely carboxyl group functionalized one, exhibited maximum lead adsorption capacity of 298.49 mg g^−1^ which was significantly higher than that of the best performing MWCNT sample (amino-functionalized MWCNT, 58.547 mg g^−1^).

## Abstract 2

Functionalization with same functional group results in varied lead adsorption performance for different nanostructured carbon materials.

## Graphical abstract

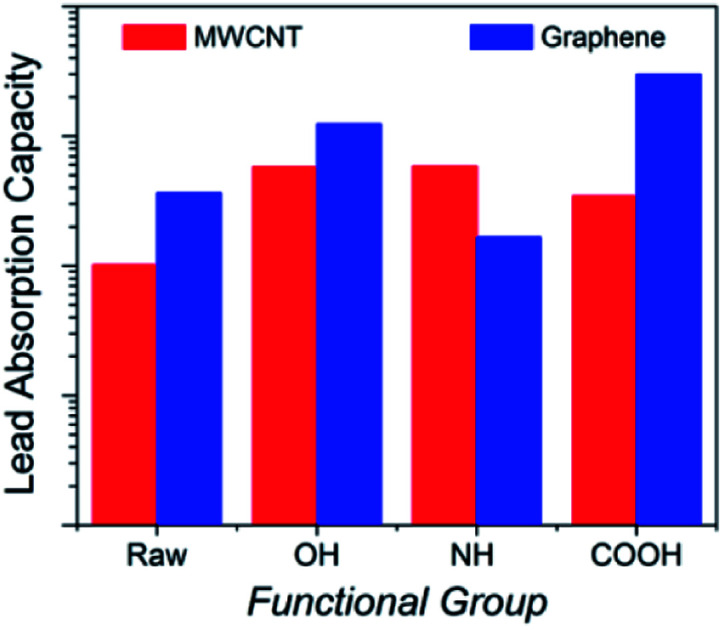

## Introduction

Carbon based nanomaterials are of significant interest for environmental applications, such as photocatalysis and pollutant removal in general. Consequently, they have been extensively studied, alone or in various composites.^1–54^ Among various pollutants, the removal of heavy metals from water is of considerable interest due to low adsorption capacity of conventional adsorbents (active carbon, polymers) for heavy metal removal.^1^ Due to their high surface areas, tunable surface chemistry, and large capacity for contaminant adsorption, carbon based nanomaterials are of particular interest for this application.^1,44^ Various carbon based nanomaterials have been demonstrated for heavy metal removal, including Pb, Cu, Cd, Cr, As, Co, Ni, and Hg.^42,44–48^ Heavy metals are known to have harmful effects on human health even at trace level concentrations,^43,44^ and among these lead pollution is considered as one of the most significant concerns^44^ since it is widespread in the environment.^13^ Exposure to Pb can result in damage to central nervous system, renal, gastrointestinal, hematopoietic, cardiovascular, and reproductive systems^43,45^ and brain function.^43^

It is well known that surface functionalization plays an important role in heavy metal removal,^1,44^ since it improves the hydrophilicity of the nanocarbon, and it can also enable increased adsorption of the target contaminant *via* electrostatic interactions.^1^ In the case of Pb^2+^, it was reported that the adsorption mechanisms include interaction with oxygen containing and/or negatively charged functional groups, cation–π interaction, and adsorption on defect sites.^1^ In general, physisorption, (ion exchange, electrostatic interaction) and chemisorption (surface complexation) processes coexist.^16^ In some cases, specific mechanisms have been found to be dominant. For example, surface complexation rather than ion exchange was identified as main adsorption mechanism for lead for oxidized MWCNTs,^26^ while another study identified that the dominant mechanism of lead adsorption was chemisorption involving phenolic groups.^25^ In the case of graphene oxide (GO), it was proposed that hydroxyl and carboxyl groups located at the edges of GO sheets were main participants in lead ion complexation.^38,41^ It is not clear, however, whether those findings are generally applicable due to large variation in reported experimental conditions for lead removal.

Furthermore, while the carbon nanomaterials typically display higher adsorption capacity compared to conventional adsorbents, the reported results on the adsorption affinity of divalent heavy metals can be contradictory.^1^ In addition, for some materials such as graphene oxide (GO), very wide range was reported for Pb^2+^ adsorption capacity (36–789.9 mg g^−1^).^1,41^ Part of the reason for that could be differences in the synthesis method of GO, which is known to affect the adsorption capacity.^8^ Other reasons may include differences in pH, ionic strength, and experimental conditions in general (contact time, mixing rate, *etc.*).^1^ Thus, direct comparisons of different materials from the published data would be difficult. Nevertheless, it is very clear that surface functionalization and/or preparing nanostructured carbon-based composites can result in efficient heavy metal removal from water. A wide range of surface functionalizations, morphologies, and different composites has been reported,^5–29^ including those based on carbon nanotubes (CNTs),^5–7,10–13,15–17,23–26,37,40^ graphene oxide (GO),^8,9,14,18–22,27,30,31,36,38–41^ and reduced graphene oxide (RGO).^34^

It is well recognized that –COOH and –OH functional groups result in negative surface charge in aqueous solution, which enhances the adsorption of positively charged metal ions.^1,10,38,41^ Carboxylic group functionalization in particular was found to result in high efficiency of Pb removal by multiwall carbon nanotubes (MWCNTs) and graphene oxide.^13,20,38,41^ Similar finding, *i.e.* significant enhancement of lead adsorption, has also been reported for amino group, and the contribution of functional groups to the total lead adsorption was estimated to exceed 80%.^16^ However, molecular dynamics simulations of single wall CNTs functionalized with carboxyl, hydroxyl, and amide (–CONH_2_) functional groups predicted that an improvement in lead adsorption with carboxyl functionalization is significantly higher compared to hydroxyl or amide groups.^17^ Thus, there is an obvious interest in conclusively establishing what type of functional groups and what type of nanostructured carbon will yield superior lead adsorption performance by experimental investigation, before proceeding with more complex functionalization and/or composite syntheses.

While there have been comparisons of lead adsorption performance as-prepared, oxidized and amine-functionalized MWCNTs,^16^ the comparisons of different functional groups on different forms of nanostructured carbon have been lacking. Here we compare the lead adsorption on MWCNTs and reduced graphene oxide (RGO) for samples without functionalization, as well as samples functionalized with hydroxyl, carboxyl, and amino group. The samples have been commercially obtained, since commercial samples are relevant for practical use. In addition, we have prepared graphene oxide (GO) samples by modified Hummers' method, and compared the performance to different commercial RGO samples. All the samples have been comprehensively studied, and their performance in lead adsorption has been compared not only in terms of adsorption kinetics and isotherms but also in terms of lead removal by filtration. Differences in the performance for amino-group functionalization for MWCNT and RGO are discussed, since amino-group functionalization improved the lead adsorption capacity of MWCNTs, but reduced the lead adsorption of RGO. Unlike amino-group, hydroxyl- and carboxyl-groups result in performance improvements regardless of the type of nanostructured carbon. Thus, these functional groups would be most relevant for the preparation of composite materials for practical applications.

## Experimental

### Chemicals and materials

Graphite (325 mesh) was purchased from Aladdin. Potassium permanganate (KMnO_4_ AR), ferric chloride (FeCl_3_ 99%), and sodium hydroxide (NaOH AR) was purchased from Dieckmann. Lead(ii) nitrate (Pb(NO_3_)_2_ 99%) was purchased from Alfa Aesar. Sulphuric acid (H_2_SO_4_ AR) was purchased from RCI Labscan. Hydrogen peroxide (H_2_O_2_ 30%), hydrochloric acid (HCl 37%), and nitric acid (HNO_3_ 68%) were purchased from VWR Chemical. Phosphoric acid (H_3_PO_4_ 85%) was purchased from Lancaster Synthesis. RGO and carbon nanotubes with different functionalizations were purchased from Times Nano. The properties of different nanostructured carbon samples are summarized in ESI, Tables S1 and S2.† Morphologies of different samples have been characterized by scanning electron microscopy (SEM). Obtained images are also shown in ESI, Fig. S1 and S2.†

### Synthesis of GO-HM

GO-HM was prepared using a modified Hummers' method. Initially, 1.5 g of graphite was mixed with 9.0 g of KMnO_4_. In a separate round bottom flask, 180 mL of H_2_SO_4_ was mixed with 20 mL of H_3_PO_4_ in an ice bath. The solid mixture was then slowly added to the acid in an ice bath, producing a deep green solution. The mixture was then heated at 50 °C in an oil bath for 2 days. The solution was poured into a beaker containing 200 g of ice and 3 mL of 30% H_2_O_2_. The obtained solid was centrifuged and washed twice with water, HCl, and ethanol, respectively. The obtained brown solid was dried at 70 °C in vacuum.

### Kinetic studies

10 mg of sorbent was placed in a 20 mL glass vial, and then 10 mL of 50 ppm Pb^2+^ solution was added to the vial and stirred with a magnetic stir bar for a specified time (1 min, 2 min, 3 min, 4 min, 5 min, 10 min, and 15 min). The solution was then filtered by 0.22 μm syringe filter, and the filtrate was collected for ICP-OES analysis.

### Adsorption isotherm studies

10 mg of sorbent was placed in a 20 mL glass vial. For each vial, 10 mL, 25 ppm, 50 ppm, 100 ppm, 250 ppm, and 500 ppm Pb^2+^ solution was added to the vial and stirred with a magnetic stir bar for 90 minutes. The solution was then filtered by 0.22 μm syringe filter, and the filtrate was collected for ICP-OES analysis.

### Inline filtration experiment

Laboratory wipes was first cut into 5.5 cm × 5.5 cm pieces. One piece was pressed into a cone with about 5 mm height and pushed into a 1 mL pipette tip. The sorbent was then loaded into the pipette tip. Finally, another piece was pressed into a ball and pushed into the pipette tip covering the sorbent. 50 mL of 10 ppm Pb^2+^ solution was then allowed to flow through the filter under vacuum. After that, one extra mL of the feeding solution was passed through the filter and was collected for ICP-OES analysis.

### Analysis and characterization

Lead ion content in water was determined by Inductively Coupled Plasma Optical Emission Spectroscopy (ICP-OES) measurements using PE Optima 8300 ICP-OES. Scanning electron microscopy (SEM) images were taken by Hitachi S4800. Fourier transform infrared spectroscopy (FTIR) measurements were performed using a PerkinElmer Spectrum Two IR Spectrometer. XPS was performed using an EscaLAB 205 xi XPS system equipped with a Al Kα X-ray source. Brunauer–Emmett–Teller (BET) surface areas of the samples were characterized by a Quantachrome QUADRASORB EVO analyzer at −195.8 °C.

## Results and discussion

The experimental data of lead adsorption on different samples are modeled with Freundlich and Langmuir isotherm models.^3,7,13^ The Langmuir model assumes that the adsorption is localized in a monolayer and that there is no interaction among adsorbed species, and describes the adsorbed quantity per quantity of sorbent material *q*_e_ as:^3,13^1
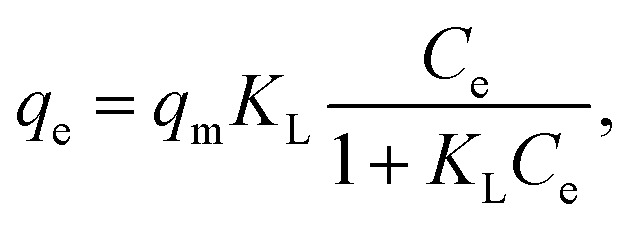
where *q*_m_ is the maximum adsorption capacity, *C*_e_ is the equilibrium concentration, and *K*_L_ is the enthalpy of adsorption.^3^ This equation can be approximated as:^13^2
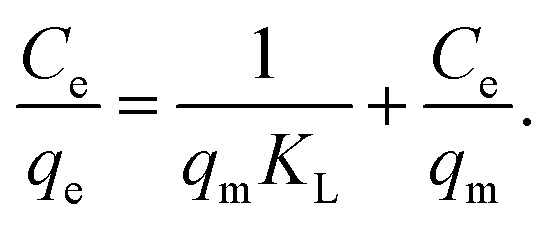


Freundlich model is an empirical model which assumes the involvement of different sites with several adsorption energies:^3,7,13^3
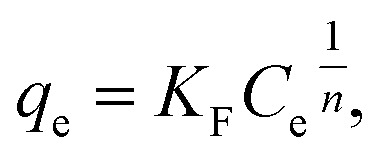
where *K*_F_ is a Freundlich constant,^3,7^ and *n* is a constant related to isotherm nonlinearity.^7^Eqn (3) can be either fitted directly or rewritten in a linear form as follows:4
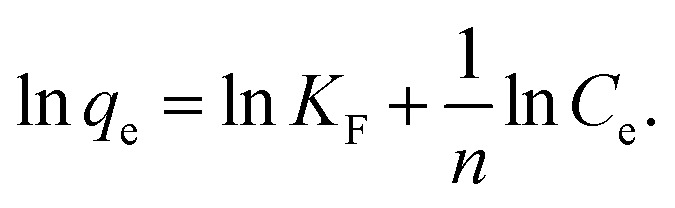


Linear fitting to eqn (2) was used to fit the data to Langmuir isotherm model, while nonlinear fitting for eqn (3) was used to fit the data to Freundlich adsorption isotherm model. Obtained results are shown in Tables 1 and 2, respectively, as well as Fig. S3 in ESI.† It can be observed that for all GO and RGO samples good fit to Langmuir model is obtained, with *r*^2^ values ranging from 0.972 to 0.996. This implies that in these samples, inherent assumptions of the model are fulfilled, *i.e.* that the monolayer coverage is achieved and that there is no interaction between the adsorbates.^13^ Obtained *q*_m_ values indicate that GO-HM, OH-RGO, and especially COOH-RGO samples are very promising for lead adsorption, with obtained *q*_m_ values of 118.42 mg g^−1^, 123.76 mg g^−1^ and 298.49 mg g^−1^, respectively. Surprisingly, NH-RGO sample shows even worse performance compared to RGO samples, indicating that amino-group functionalization does not enhance lead adsorption on RGO. The obtained values of maximum adsorption capacity fall into the previously reported range for GO between 36 mg g^−1^ to 659 mg g^−1^.^1^ Different from high lead adsorption capacity previously reported for amino-functionalized graphene of 461 mg g^−1^,^4^ the lowest maximum adsorption capacity in our work is obtained for NH-RGO samples. However, obtained result is in agreement with a report that GO-NH_2_ had lower lead adsorption capacity compared to GO.^3,30^ Furthermore, good fit to Freundlich model is obtained for RGO and GO-HM samples, while the obtained *r*^2^ values are lower for RGO samples with different functional groups (–OH, –COOH, –NH_2_). Better fit to a Langmuir isotherm (higher correlation coefficient values) compared to Freundlich isotherm is in agreement with a previous report on the adsorption of metals on GO nanosheets.^3,30,38^ It can indicate that the adsorption surfaces of OH-RGO, COOH-RGO, and NH-RGO are likely homogeneous, and it also indicates a possible chemical adsorption process,^30,38^ which would be expected for the interaction between the functional groups and lead ions.

**Table 1:** Isotherm fitting results for MWCNT samples. Correlation coefficient *r*^2^ values are given for comparison of goodness of fit

Model		MWCNT	OHCNT 1.8%	OHCNT 5.5%	NHCNT	COOH CNT
Langmuir (linear)	*r* ^2^	0.795	0.972	0.982	0.941	0.759
	*q*_m_ (mg g^−1^)	10.167	12.099	57.904	58.547	34.545
	*K*_L_	0.009	0.025	0.027	0.020	0.008
Freundlich	*r* ^2^	0.775	0.972	0.968	0.929	0.811
	*n*	2.218	2.844	3.046	2.936	2.08
	*K*_F_ (mg g^−1^)	0.555	1.543	7.604	6.726	1.51

**Table 2:** Isotherm fitting results for RGO and GO samples. Correlation coefficient *r*^2^ values are given for comparison of goodness of fit

Model		RGO	OH-RGO	NH-RGO	COOH-RGO	GO-HM
Langmuir (linear)	*r* ^2^	0.972	0.993	0.996	0.995	0.989
	*q*_m_ (mg g^−1^)	36.496	123.76	16.667	298.49	118.42
	*K*_L_	0.021	0.062	0.085	0.330	0.06
Freundlich	*r* ^2^	0.965	0.746	0.852	0.811	0.977
	*n*	3.013	9.601	4.925	7.337	3.466
	*K*_F_ (mg g^−1^)	4.482	61.401	5.110	138.81	21.698

In the case of MWCNT, we can observe that good fit to both adsorption isotherm models is obtained for all the samples except MWCNT and COOH-CNT. The CNT-based samples in general exhibit rather poor dispersion in water, which is the likely reason for inferior fit of the data despite stirring of the solution. RGO-based and GO-HM samples exhibit significantly better dispersion in water, as shown in Fig. S4 and S5.† The obtained maximum adsorption capacity is lower than that previously reported for MWCNT (16.9 mg g^16^ and 29.9 mg g^−1^), while the obtained value for amino-functionalized MWCNT samples is in good agreement with a previous report for diethylenetriamine-functionalized MWCNTs (58.26 mg g^−1^).^16^ It was also previously reported that the maximum adsorption capacity of oxidized MWCNT was 17.5 mg g^−1^, which was slightly higher than that of MWCNT 16.9 mg g^−1^.^16^ Generally, lead adsorption in MWCNTs increases with increased oxygen content.^25^ This is in agreement with the obtained increase in the maximum adsorption capacity for higher OH-group content of MWCNT samples, so that the performance of 5.5% OHCNT is comparable to that of NHCNT samples.

Adsorption kinetics, which provides information on the adsorbate uptake rate, was also studied. Pseudo-second order kinetic model is commonly used to describe kinetic data for lead adsorption on nanostructured carbon samples, and it is given by a following equation:^3,7,13,16,21,25,38^5
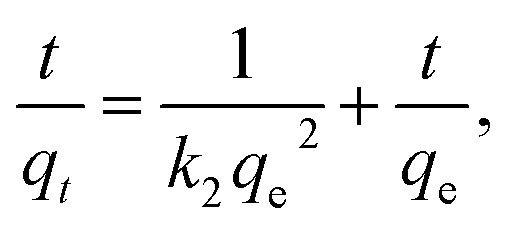
where *k*_2_ is the rate constant of the adsorption, and *q*_e_ is the equilibrium adsorption capacity, *q*_*t*_ is adsorption capacity at a time, and *t* is time. For all the samples, excellent fits to the pseudo-second order kinetic model are obtained, as shown in Tables 3 and 4 and Fig. S6 in ESI.† It can be observed that the equilibrium has been reached in all the samples. The obtained equilibrium adsorption capacity values follow the same trends as the estimated maximum adsorption capacities from Langmuir model for adsorption isotherms. The highest capacities are obtained for OH-CNT and NH-CNT among MWCNT samples, while for RGO/GO samples the highest capacities are obtained for COOH-RGO and OH-RGO, and closely followed by GO-HM. To comprehensively test the ability of the materials to remove lead from aqueous solutions, in-line filtration experiment has also been performed, and the obtained results are given in Table 5. The experiment could not be conducted for GO-HM samples due to the clogging of the filter. The different RGO samples follow the same trends as expected from adsorption isotherms and kinetics measurements, with COOH-RGO and OH-RGO exhibiting excellent performance, and NH-RGO performing worse than non-functionalized RGO. Among MWCNT samples, MWCNT and OH-CNT with lower OH-group concentration (1.8%) had significantly worse performance compared to remaining CNT samples.

**Table 3:** Kinetic fitting results for different MWCNT samples. Correlation coefficient *r*^2^ values are given for comparison of goodness of fit

Parameter	MWCNT	OHCNT 1.8%	OHCNT 5.5%	NHCNT	COOH CNT
*r* ^2^	0.9975	0.9974	0.9975	0.9993	0.9979
*q*_e_	4.2050	4.6402	22.8102	19.8728	7.3981
*k*_2_	5.8971	1.5476	0.1996	0.0797	7.0004

**Table 4:** Kinetic fitting results for RGO and GO samples. Correlation coefficient *r*^2^ values are given for comparison of goodness of fit

Parameter	RGO	OH-RGO	NH-RGO	COOH-RGO	GO-HM
*r* ^2^	0.9743	1	0.9945	1	0.9934
*q*_e_	11.8319	49.2126	6.9667	49.7512	37.3134
*k*_2_	0.2585	0.688171	0.152124	0.17956	0.0325

**Table 5:** In-line filtration results for different samples. Flow rate is also given

Sorbent	Flow rate (mL min^−1^)	% Removal
MWCNT	3	14.32 ± 7.48
OH-CNT 1.8%	3	12.66 ± 4.96
OH-CNT 5.5%	3.3	32.90 ± 1.69
NH-CNT	3.3	28.03 ± 4.18
COOH-CNT	4	25.43 ± 2.79
RGO	6	24.97 ± 5.86
OH-RGO	2	76.16 ± 3.61
NH-RGO	2	13.79 ± 2.73
COOH-RGO	4	77.31 ± 0.65

**Fig. 1:**
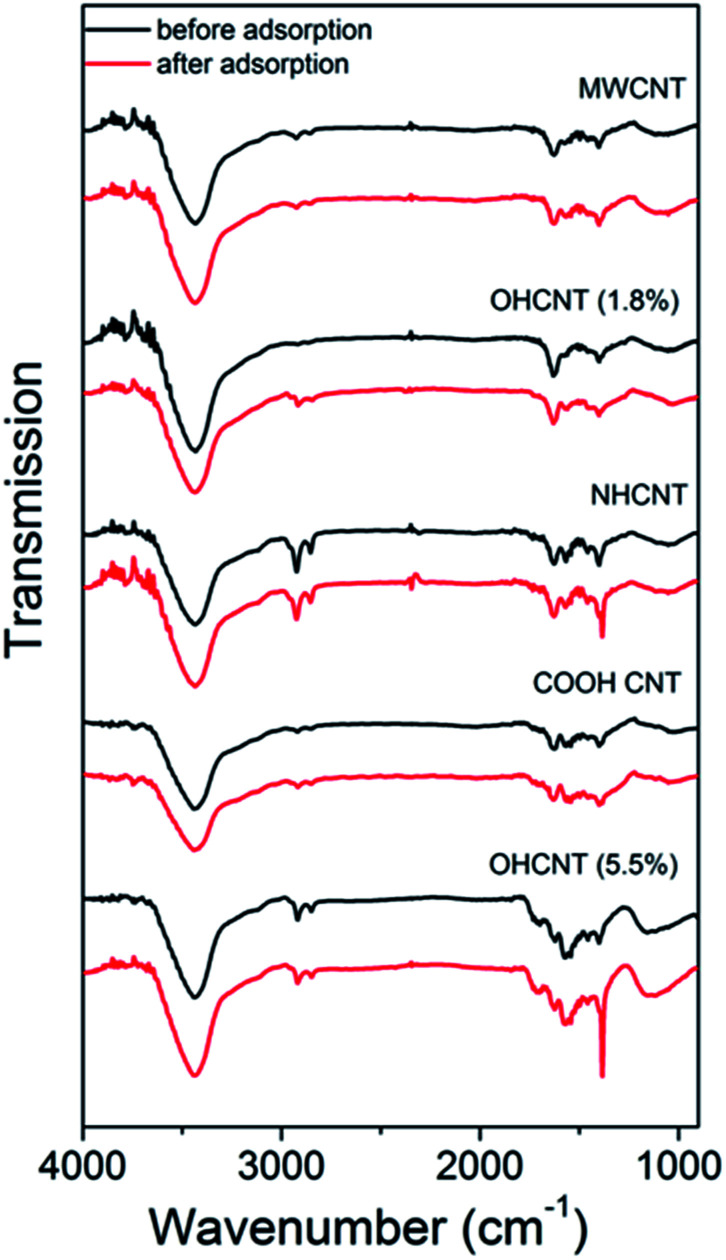
FTIR spectra of MWCNT samples with different functionalizations before and after lead adsorption.

**Fig. 2:**
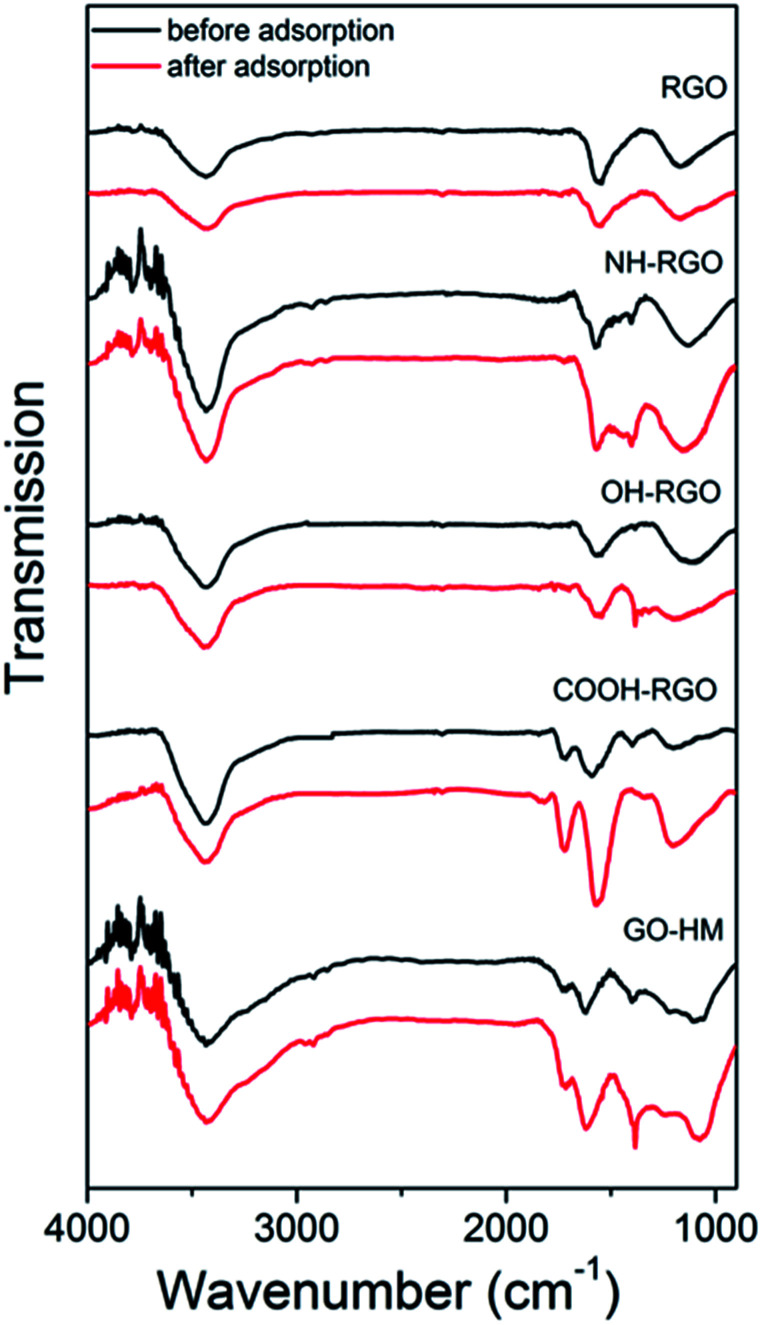
FTIR spectra of RGO/GO samples with different functionalizations before and after lead adsorption.

Thus, we can consistently observe worsening of the lead adsorption for RGO samples with amino-functionalization, while the lead adsorption is significantly improved in amino-functionalized MWCNT. To investigate the adsorption mechanisms in more detail, FTIR measurements have been performed, and the obtained results are shown in Fig. 1 and 2. The FTIR spectra of the samples exhibit expected peaks at ∼3400 cm^−1^ due to OH groups,^13,23,29,38^ peaks attributed to –CH_2_ and CH_3_ vibrations at 2948 cm^−1^ and 2848 cm^−1^,^7,23,29^ and 1716–1726 cm^−1^ C=O vibration in COO–.^8,20,29,38^ Other commonly observed peaks in nanostructured carbon (CNT or GO) include vibrations attributed to C=C and C=O carbonyl groups 1620–1634 cm^−1^,^8,20,29,38^ C–O–C and C–O groups at 1217–1220 cm^−1^ and 1047–1050 cm^−1^,^8,20,38^ C–O stretching 1097 cm^−1^,^29^ 1373 cm^−1^ C–O carboxyl vibration,^8^ deformation of C–H bond at 1450 cm^−1^,^7^ and tertiary C–OH groups deformation at ∼1400 cm^−1^.^38^ Samples containing amine groups may also exhibit amide carbonyl stretching at 1650 cm^−1^,^29^ 1580 cm^−1^ and 1180 cm^−1^ N–H in plane and C–N bond stretching,^29^ 1117 and 1052 cm^−1^ C–N bond stretching,^7^ and 1630 and 1587 cm^−1^ bending vibrations of N–H.^7^

In general, the comparisons of FTIR spectra of samples before and after lead adsorption can result in observations of changes in peak intensity, peak shifts and the appearance or disappearance of peaks.^16^ For example, the interactions with carboxyl group can lead to the changes in intensity of the peaks at 1726 cm^−1^ and 1260 cm^−1^,^16^ as well as peaks at 1635 cm^−1^ and 1384 cm^−1^ which correspond to asymmetric and symmetric COO– vibrations.^16^ Changes in the peak at 1097 cm^−1^ corresponding to phenol group are also sometimes observed.^16^ In addition to the interaction with carboxyl group, lead adsorption can result in the shifts of stretching vibrations of amino- and hydroxyl groups.^12^ In amino-group functionalized samples, shifts of the peaks at 1650 cm^−1^ and 1580 cm^−1^ corresponding to amide I and N–H in plane stretching have been reported, as well as changes in the intensity of bands corresponding to C–N stretching and out-of-plane NH_2_ bending mode at 1180 cm^−1^ and 800 cm^−1^.^16^ It was previously reported that the lead adsorption resulted in the reduction if C–O stretching vibration at 1280 cm^−1^, as well as 1647 cm^−1^ peak in 1640–1750 cm^−1^ band corresponding to C=O vibrations, while hydroxyl peak did not exhibit significant changes, indicating stronger interaction between lead and carboxylic and/or carbonyl groups compared to hydroxyl groups.^9^

The obtained FTIR spectra are shown in Fig. 1 for CNT-based samples and Fig. 2 for GO-based samples. It can be observed that in nanotube samples with lower lead adsorption capacity (MWCNT, OH-CNT 1.8%, COOH-CNT) there are no prominent changes in the FTIR spectra. For OH-CNT 1.8% sample we can see an increase in the peak at ∼1400 cm^−1^, and such an increase is considerably more pronounced in OH-CNT 5.5% sample, indicating that in these samples there is an interaction between lead ions and hydroxyl groups, resulting in an increase of vibration attributed to C–OH groups deformation at ∼1400 cm^−1^.^38^ This is consistent with physisorption^16^ being dominant mechanism on CNT-based samples, with the exception of those containing –OH groups where chemisorption related to hydroxyl groups can be observed. In NH-CNT samples, no significant changes are observed, other than small increase in the peak close to ∼1400 cm^−1^.

On the other hand, in GO-based samples exhibiting high performance, such as COOH-GO we can observe a more significant change in the FTIR spectra, with prominent increase in features at ∼1600 cm^−1^ and ∼1200 cm^−1^, which can be attributed to carbonyl and C–O vibrations.^8,20,29,38^ In OH-RGO, NH-RGO, and GO-HM samples, we observe an increase corresponding to C–OH groups deformation at ∼1400 cm^−1^,^38^ while NH-RGO also exhibits an increase in the feature at ∼1160 cm^−1^ which can be attributed to C–N stretching.^16^ A prominent increase in the wavenumber range of 1050–1100 cm^−1^ is observed for GO-HM samples, which is probably assigned to C–O groups. Thus, we can observe that amine functionalization in RGO samples results in an obvious interaction between lead ions and the amino groups, while no such feature is observed in NH-CNT samples. Thus, the adsorption mechanism of lead ions on functionalized carbon nanostructures is dependent not only on the functional group but also on the morphology of the nanocarbon, which may affect the accessibility of the functional groups (for example carboxyl and hydroxyl groups at the edges of GO sheets were found to interact strongly with lead ions^38^). Similar trends in terms of the effect of nanocarbon morphology, *i.e.* improved adsorption performance of samples containing GO compared to those with CNTs, were previously observed in Cr(vi) removal, although the mechanism has not been clarified.^50^

**Fig. 3:**
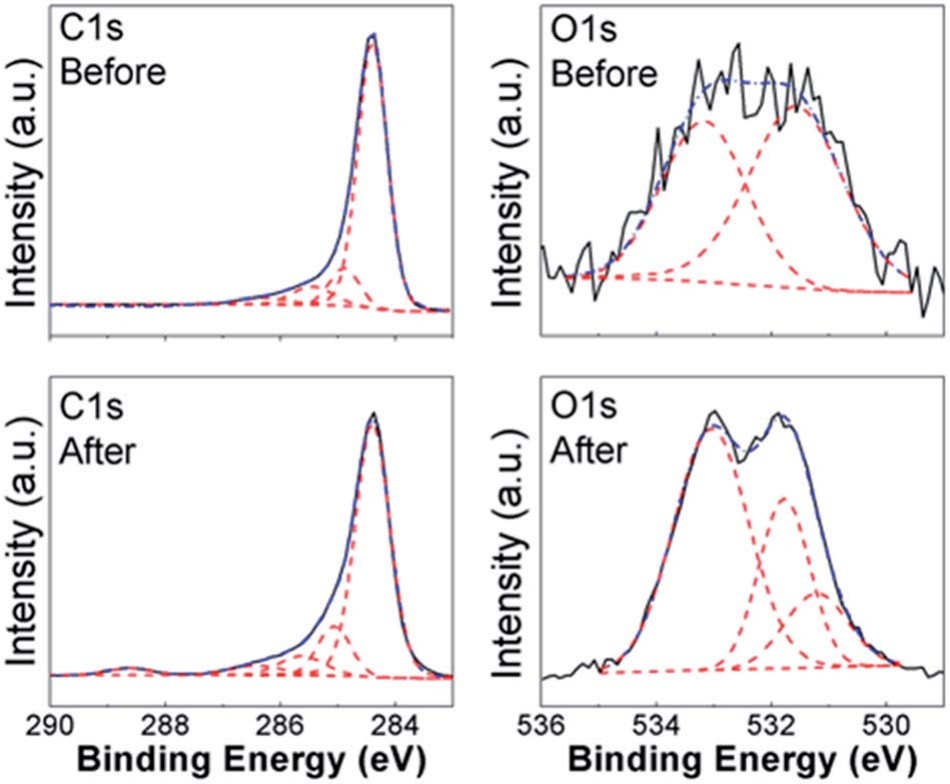
XPS spectra of C 1s and O 1s peaks of COOHCNT samples before and after lead adsorption.

**Fig. 4:**
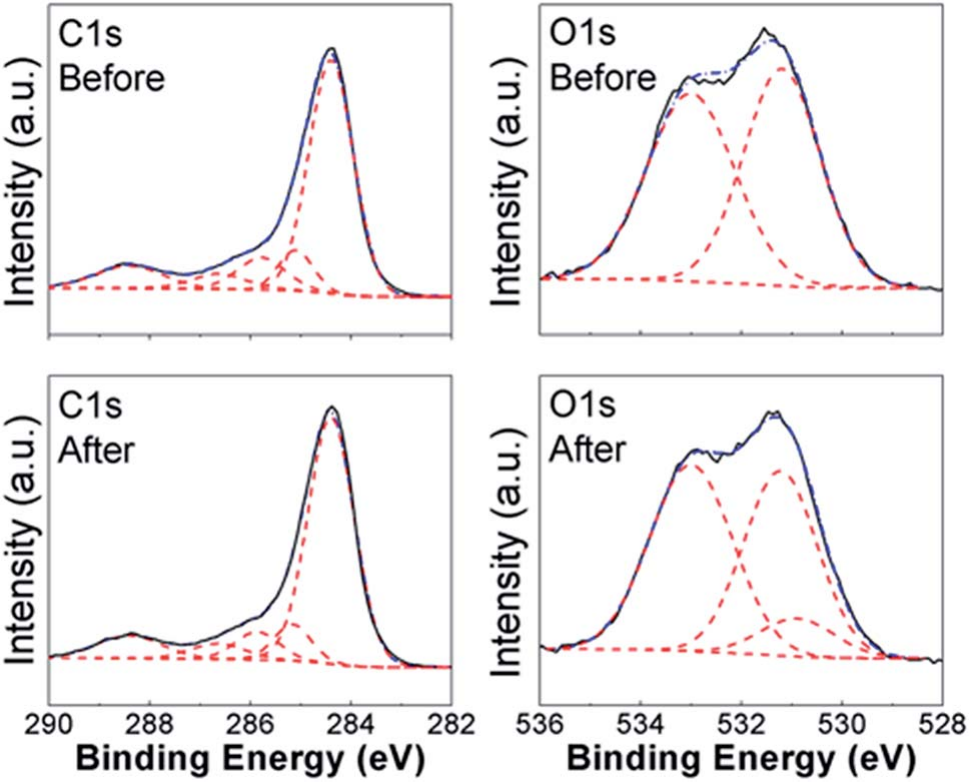
XPS spectra of C 1s and O 1s peaks of COOH-RGO samples before and after lead adsorption.

To obtain further insights into the lead adsorption process, XPS measurements have been conducted. The obtained results are summarized in Fig. 3 and 4 for COOH functionalized CNT and RGO samples, respectively, as well as ESI, Tables S3 and S4, and Fig. S7–S15.† In all the samples, we can observe multiple components in the C 1s and O 1s peaks. Different contributions to C 1s peak are assigned as follows: 284.4 eV to C=C bonding, 285.1–285.4 eV to C–C bonding, 285.8–286.4 eV to C–O bonding, 286.8–287.6 eV to C=O bonding, and 288.8–289.5 eV to O–C=O bonding.^8,14,20,27,30,34,39,55–60^ For O 1s peak, peak at 533.1–533.6 eV corresponds to C–OH bonding, while peak at 530.6–532.4 eV corresponds to carboxyl (–COO) and carbonyl (C=O) bondings.^27,30,37,56–58^ In addition, Pb signal was detected in all the samples after the lead adsorption (see ESI, Fig. S15†). In the CNT samples containing the COOH and OH functional groups, we can observe changes in the C=O, C–OH, and O–C=O bondings in C 1s and O 1s spectra. This is in agreement with the FTIR results, and previous literature reports that oxygen-containing functional groups on the surface of nanostructured carbon play a role in the metal adsorption.^26,27^ Some changes are also observed in the relative intensities of peaks corresponding to C–C and C=C bondings, which is consistent with physisorption. It should also be noted that in CNT-based samples containing OH and/or COOH groups significant change occurs in the peak corresponding to O–C=O bonding which appears after adsorption, while in the corresponding RGO/GO-based samples the peak corresponding to O–C=O bonding is present both before and after lead adsorption. Similar to CNT samples with carboxyl and/or hydroxyl groups, we also observe similarities in the behavior of RGO samples containing COOH and OH groups. While the changes in C 1s peak of GO/RGO samples are less obvious compared to CNT samples, both RGO and CNT samples with carboxyl and hydroxyl groups exhibit appearance of an additional peak in O 1s spectra at ∼530.9 eV, which can be assigned to Pb–O bond.^57,58^ Different from RGO samples, GO-HM samples exhibit very prominent peaks corresponding to both C=C and C–O bondings (Fig. S13, ESI†), and the change in the C 1s peak shape after lead adsorption can be observed, in agreement with a previous report.^8^ Unlike the samples containing hydroxyl and carboxyl functional groups, the samples without surface functionalization exhibited small changes after lead adsorption, and a weak signal corresponding to Pb 4f was detected. Thus, the obtained results provide further support that oxygen containing functional groups provide a significant contribution to lead adsorption *via* chemical interaction with Pb^2+^. Indications of this are present in both CNT-based and GO/RGO-based samples, although the trends in the observed changes do exhibit geometry dependence (for example, changes in O–C=O bondings in C 1s spectra are more significant in CNT-based samples). Despite the fact that COOH-RGO exhibited the highest lead adsorption capacity based on Langmuir model isotherm fitting, no distinct mechanisms compared to other COOH- and OH-containing samples can be observed. Thus, we can conclude that the presence of oxygen containing functional groups enhances the lead adsorption performance of all nanostructured carbon samples, with the degree of enhancement dependent on the oxygen content and sample geometry, as well as how well the samples can be dispersed in water. It should also be noted that while in CNT-based samples some correlation can be observed between BET surface area and lead adsorption, in RGO/GO-based samples there is a complete lack of correlation between the BET surface area and lead adsorption performance. Thus, we can conclude that BET surface area measurement performed based on nitrogen gas adsorption is not necessarily a good predictor of the metal ion adsorption performance in solution. It should also be noted that XPS revealed differences in behavior of NHCNT and NH-RGO samples, in agreement with FTIR results. In NHCNT samples, the N 1s peak at 399.6 eV corresponding to amino group^30^ disappears after lead adsorption, while the peak at 285.3–285.4 eV corresponding to C–N bonding^30,59^ can still be observed. In NH-RGO samples, N 1s signal can be observed both before and after lead adsorption, with small changes in peak components corresponding to imino- and amino-groups at ∼398.7 eV and ∼399.7 eV.^30,33^

## Conclusions

We investigated the lead adsorption for different types of nanostructured carbon (MWCNT, RGO) and different functional groups (hydroxyl, carboxyl, and amino-groups). We found that while amino-group functionalization significantly enhanced lead adsorption in MWCNT samples, it resulted in deterioration of the performance of RGO. On the other hand, hydroxyl and carboxyl group functionalization resulted in improved performance regardless of the starting form of carbon. The lead adsorption performance of MWCNT improved with increased number of hydroxyl functional groups, and for higher –OH content approached that of the best performing amino-functionalized samples (58.547 mg g^−1^). In the case of RGO, the best performance was obtained by COOH-RGO with maximum lead adsorption capacity of 298.49 mg g^−1^, while OH-RGO and GO samples synthesized by modified Hummers' method exhibited similar performance with maximum lead adsorption capacities of 123.76 mg g^−1^ and 118.42 mg g^−1^, respectively.

## Conflicts of interest

There are no conflicts to declare.

## Supplementary Material

RA-008-C8RA02264J-s001
