# Supplementary material for: Lead removal from water – dependence on the form of carbon and surface functionalization
Source: RSC Adv. 2018 May 18;8(33):18355–62. doi: 10.1039/c8ra02264j (PMC9080567; doi:10.1039/c8ra02264j)
Supplement: RA-008-C8RA02264J-s001 [file RA-008-C8RA02264J-s001.pdf]

Electronic Supplementary Material (ESI) for RSC Advances.
This journal is © The Royal Society of Chemistry 2018

Supporting Information

Lead removal from water – dependence on the form of carbon and surface functionalization

*Chun Sing KAM[a], Tik Lun LEUNG[a], Fangzhou LIU[a], Aleksandra B. DJURIŠIĆ[a, *], Mao Hai XIE,[a] Wai-Kin CHAN[b], Ying ZHOU[c], Kaimin SHIH[c]*

[a]Department of Physics, The University of Hong Kong, Pokfulam, Hong Kong

[b]Department of Chemistry, The University of Hong Kong, Pokfulam, Hong Kong

[c]Department of Civil Engineering, The University of Hong Kong, Pokfulam, Hong Kong

Table S1. Physical properties of different CNT samples.

Properties	MWCNT[1]	OHCNT 1.8%[2]	OHCNT 5.5%[3]	NHCNT[4]	COOHCNT[5]
**Outer Diameter/ nm**	8-15	8-15	<8	8-15	8-15
**Inner Diameter/ nm**	3-5	3-5	2-5	3-5	3-5
**Length/ µm**	10-50	10-50	10-30	~50	10-50
**Specific surface area/ m^2g^{-1}**	>90	>117	220-300	>233	>117
**Functional group content**	N/A	-OH content: 1.85 wt%	-OH content: 5.58 wt%	NH_2 content: 0.45 wt%	-COOH content: 1.28 wt%
**Synthesis method**	CVD	CVD	CVD	CVD	CVD

[1] Technical data of MWCNT purchased from Timesnano, retrieved from http://www.timesnano.com/upfile/fck/20171128/20171128_161436_20902005161629121623.pdf
[2] Technical data of OHCNT 1.8% purchased from Timesnano, retrieved from http://www.timesnano.com/upfile/fck/20171128/20171128_161447_107132222149960646l.pdf
[3] Technical data of OHCNT 5.5% purchased from Timesnano, retrieved from http://www.timesnano.com/upfile/fck/20180308/20180308_153523_19721637439418500000.pdf
[4] Technical data of NHCNT purchased from Timesnano, retrieved from http://www.timesnano.com/upfile/fck/20160623/20160623_135729_1864228545754164460.pdf
[5] Technical data of COOH CNT purchased from Timesnano, retrieved from http://www.timesnano.com/upfile/fck/20171128/20171128_161457_9913453341887644457.pdf

Table S2. Physical properties of different GO and RGO samples.

Properties	RGO[6]	OH-RGO[7]	NH-RGO[8]	COOH-RGO[9]	GO-HM
**Thickness/ nm**	0.5-3.74	0.55-3.74	0.55-3.74	0.55-3.74	2-15[a]
**Diameter/ µm**	0.5-3	0.5-3	0.5-3	0.5-3	~10[b]
**Specific surface area/ m^2g^{-1}**	500-1000	267.028[c]	99.052[c]	21.842[c]	6.545[c]
**Functional group content**	N/A	Oxygen content>10 wt%	NH_2 content: 0.5 wt%	Oxygen content>15 wt%	Oxygen content: 34.68 atomic %[d]

[6] Technical data of RGO purchased from Timesnano, retrieved from http://www.timesnano.com/upfile/fck/20161031/20161031_114930_1474592940912733661.pdf
[7] Technical data of OH-RGO purchased from Timesnano, retrieved from http://www.timesnano.com/upfile/fck/20170111/20170111_165012_1774913364808758101.pdf
[8] Technical data of NH-RGO purchased from Timesnano, retrieved from http://www.timesnano.com/upfile/fck/20170112/20170112_093206_1939678447146451758l.pdf
[9] Technical data of COOH-RGO purchased from Timesnano, retrieved from http://www.timesnano.com/upfile/fck/20160629/20160629_135923_7735295462014854825.pdf

[a] Estimated from TEM.

[b] Estimated from SEM.

[c] Obtained by BET calculation with N_2 adsorption isotherms.

[d] Obtained from XPS.

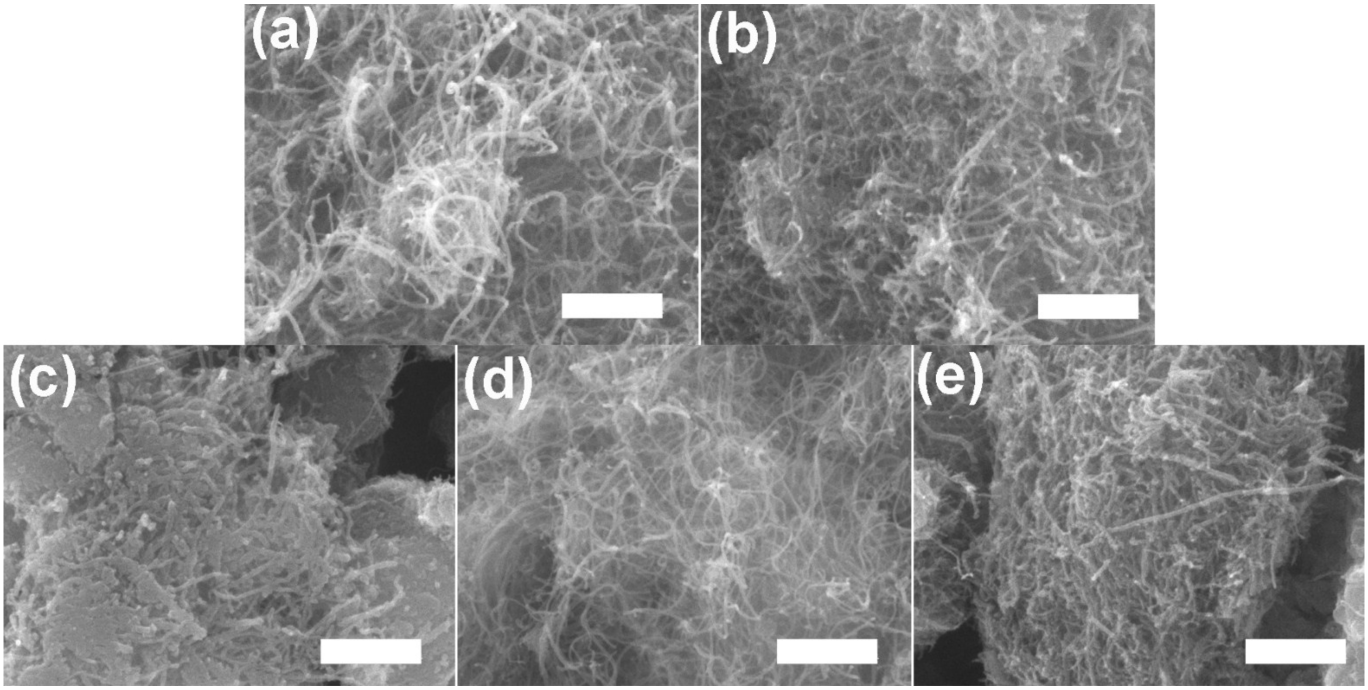

Figure S1. SEM images of CNT samples a) MWCNT; b) OHCNT 1.8%; c) OHCNT 5.5%; d) NHCNT; e) COOHCNT. Scale bar is 1 μm.

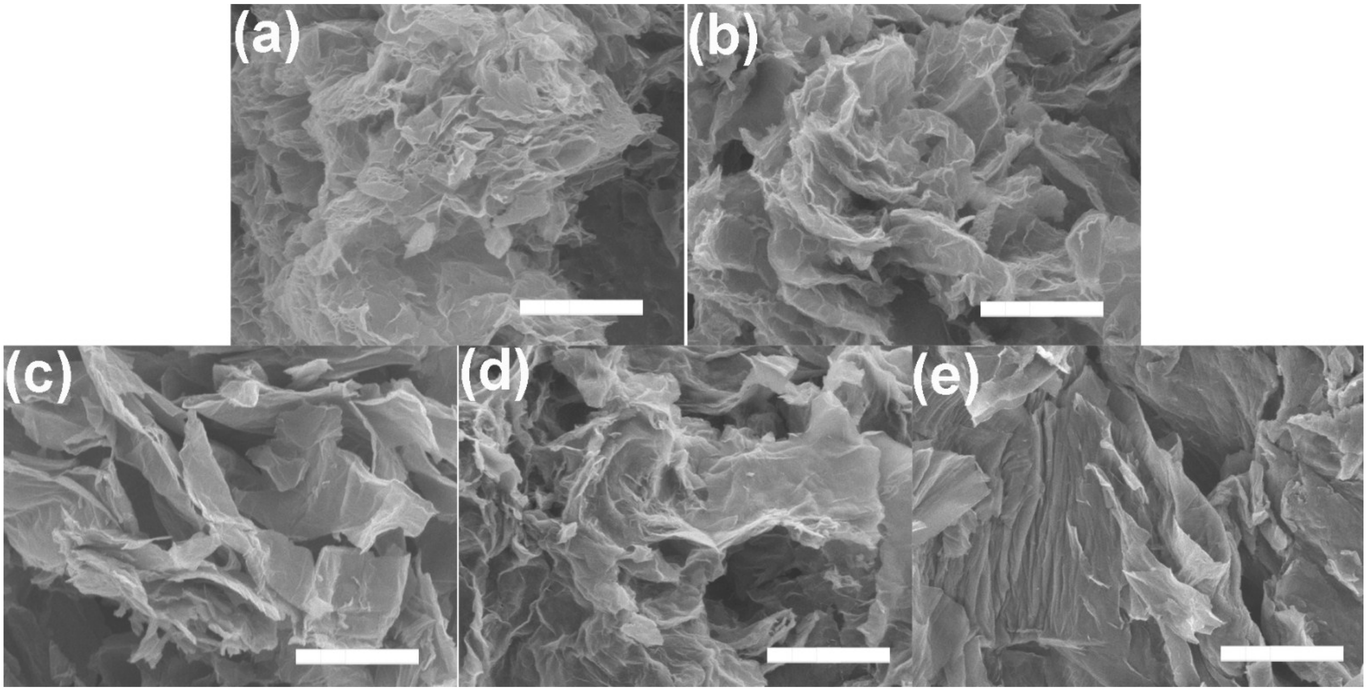

Figure S2. SEM images of RGO and GO samples a) RGO; b) NH-RGO; c) OH-RGO; d) GO-HM; e) COOH-RGO. Scale bar is 5 μm.

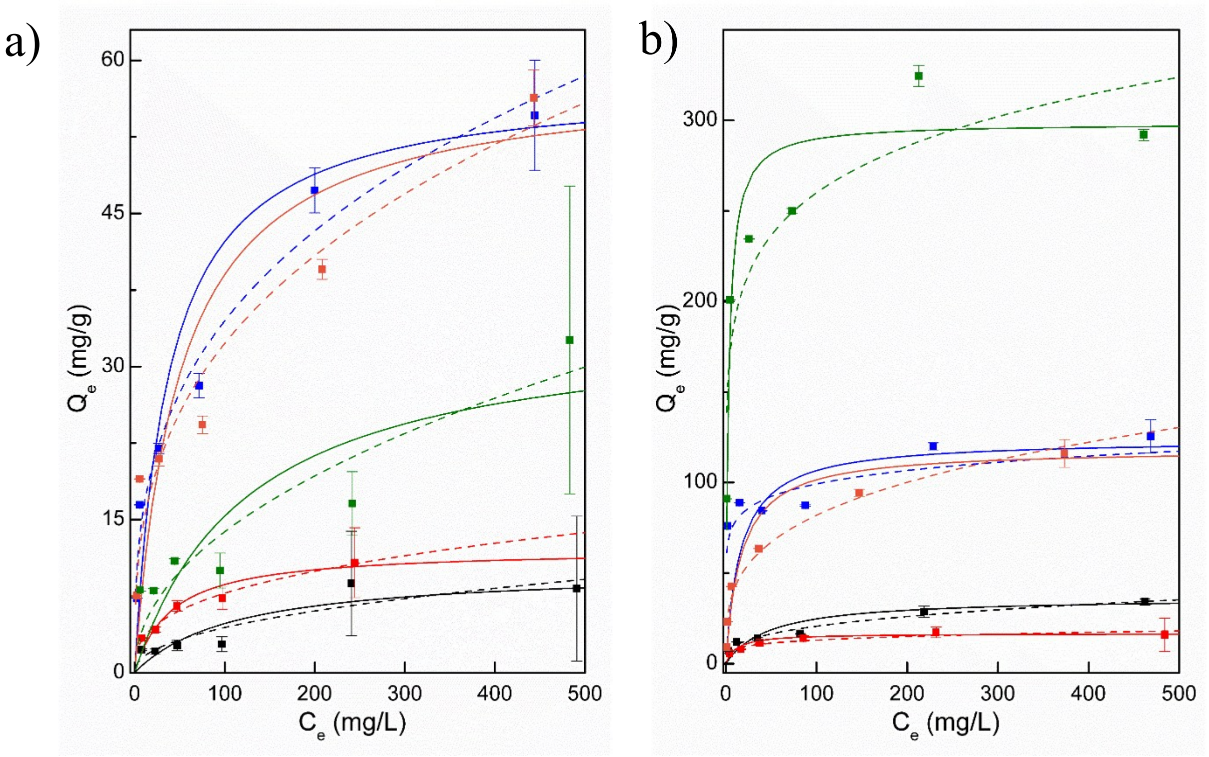

Figure S3. Adsorption isotherms of (a) CNT-based samples (Black: MWCNT; Red: OHCNT 1.8%; Blue: OHCNT 5.5%; Orange: NHCNT; Green: COOHCNT) and (b) GO/RGO-based samples (Black: RGO; Red: NH-RGO; Blue: OH-RGO; Orange: GO-HM; Green: COOH-RGO). Solid and dash lines correspond to Langmuir fitting curve and Freundlich fitting curve respectively. Measurements were done in triplicate, and the average values and corresponding standard deviation (error bars) are shown.

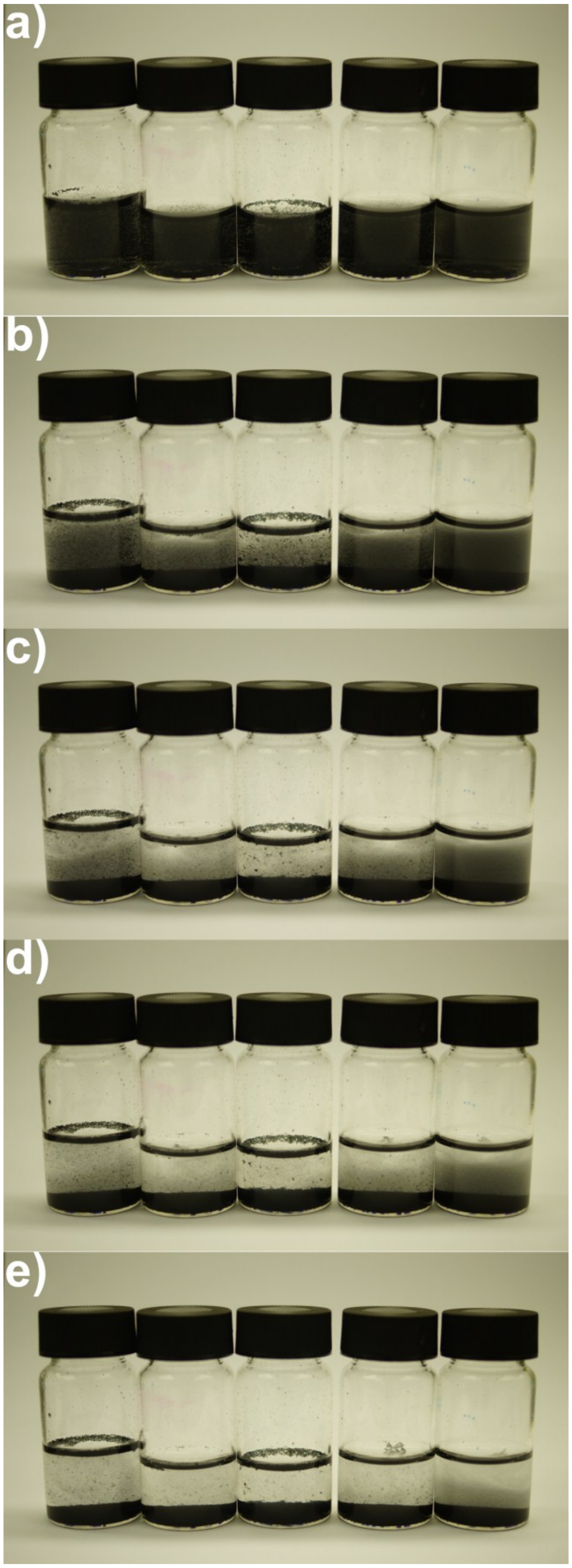

Figure S4. Photographs of different MWCNT samples (1 mg/mL) in deionized water observed at different times: a) initial, b) 1 min, c) 3 min, d) 5 min, e) 10 min. Samples from left to right correspond to MWCNT, OHCNT (1.8%), NHCNT, COOHCNT, and OHCNT (5.5%), respectively.

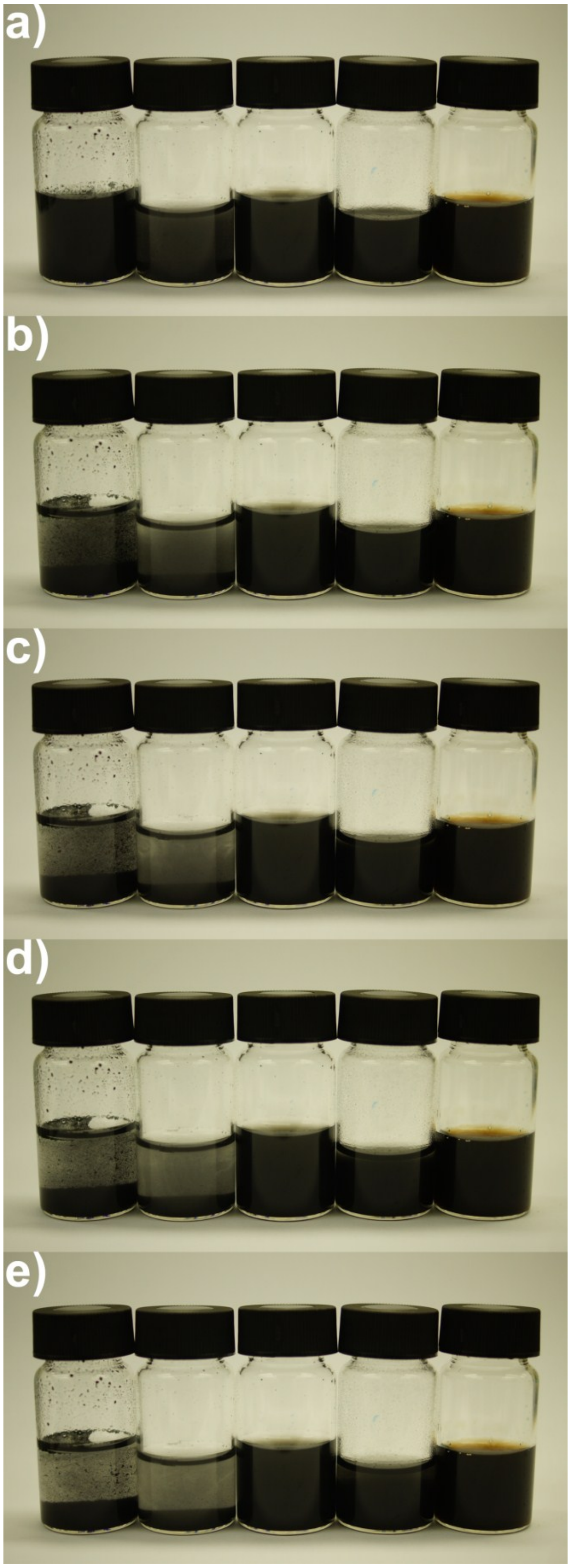

Figure S5. Photographs of different RGO and GO samples (1 mg/mL) in deionized water observed at different times: a) initial, b) 5 min, c) 10 min, d) 15 min, e) 25 min. Samples from left to right correspond to RGO, NH-RGO, OH-RGO, COOH-RGO, and GO-HM, respectively.

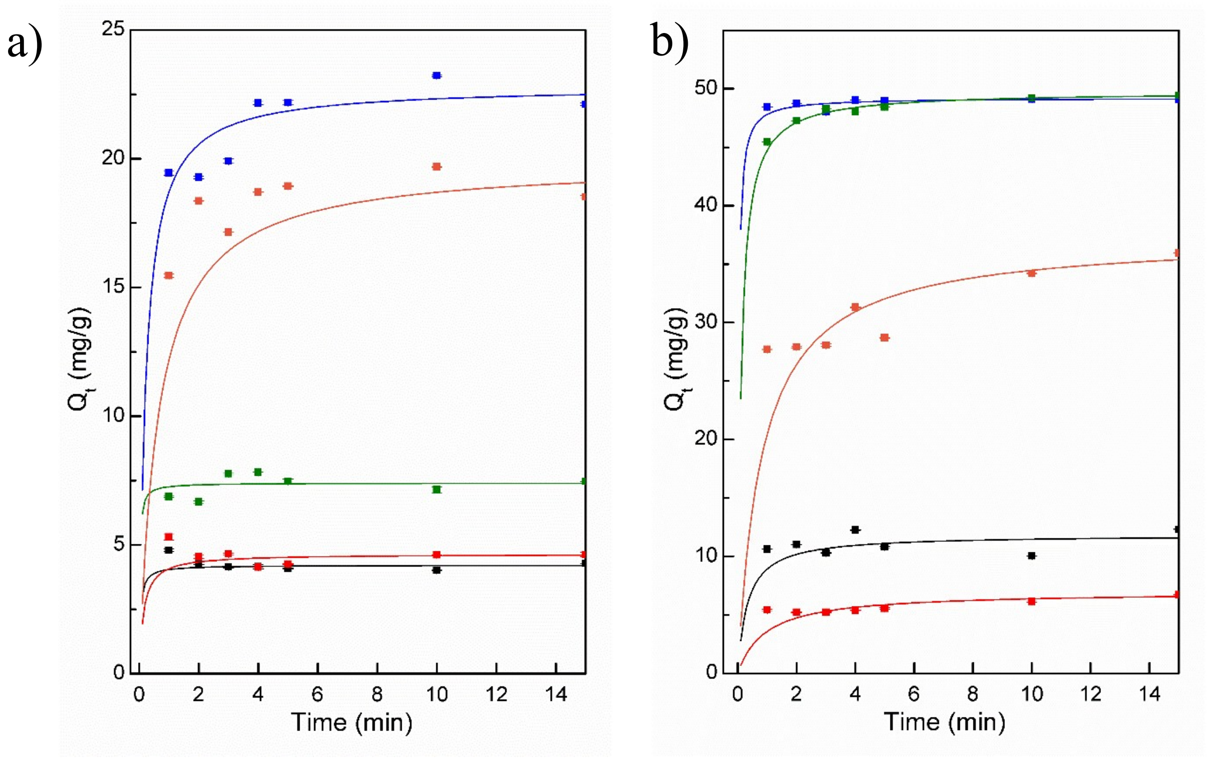

Figure S6. Kinetic fitting of (a) carbon nanotube samples (Black: MWCNT; Red: OHCNT 1.8%; Blue: OHCNT 5.5%; Orange: NHCNT; Green: COOHCNT) and (b) graphene samples (Black: RGO; Red: NH-RGO; Blue: OH-RGO; Orange: GO-HM; Green: COOH-RGO). Lines represent fitting results from pseudo-second order kinetic model. The data points are averages of two measurements.

Table S3 Summary of changes in the XPS spectra of different CNT samples before and after Pb adsorption.

Functional group		Peak position (Before to after)	% of total area (Before to after)
COOH	O1s	C=O: 531.6 eV-> 531.8eV	57.72% -> 27.09%
		C-OH: 533.2 eV-> 533.0 eV	42.28% -> 58.08%
		Pb-O: Appears at 531.2 eV.	0% -> 14.83%
	C1s	C=C: 284.4 eV-> 284.4 eV	78.82% -> 73.28%
		C-C: 284.9 eV -> 285.1 eV	10.68% -> 13.04%
		C-OH: 285.4 eV-> 285.6 eV	7.24 % ->7.10%
		C=O: 286.3 eV-> 286.4 eV	3.26% -> 4.20%
		O-C=O: Appears at 288.6 eV.	0% -> 2.37%
NH	C1s	C=C: 284.4 eV-> 284.4 eV	73.60% -> 72.23%
		C-C/C-N: 285.1 eV->285.0 eV	26.40% -> 27.77%
	N1s	C-NH_2: 399.6 eV disappears.	100% ->0%
OH(1.8%)	O1s	C=O: 531.8 eV-> 531.8 eV	54.96% ->40.39%
		C-OH: 533.2 eV -> 533.2 eV	45.04% ->56.85%
		Pb-O: appears at 530.9 eV	0% -> 2.76%
	C1s	C=C: 284.4 eV-> 284.4 eV	65.54% -> 64.35%
		C-C: 284.7 eV -> 285.0 eV	28.70% -> 22.61%
		C-OH: 285.9 eV-> 285.9 eV	7.49 % ->10.61%
		O-C=O: appears at 288.7 eV	0% -> 2.44%
OH(5.5%)	O1s	C=O: 531.4 eV-> 531.8 eV	54.02% ->14.11%
		C-OH: 533.1 eV -> 533.0 eV	45.98% ->46.10%
		Pb-O: appears at 530.9 eV	0% -> 39.79%
	C1s	C=C: 284.4 eV-> 284.4 eV	59.63% -> 71.29%
		C-C: 284.7 eV -> 285.2 eV	33.64% -> 12.69%
		C-OH: 286.2 eV-> 286.1 eV	6.73 % ->12.75%
		O-C=O: Appears at 288.6 eV.	0% -> 3.27%
None (MWCNT)	C1s	C=C: 284.4 eV-> 284.4 eV	78.47% ->79.27%
		C-C: 285.1 eV -> 285.1 eV	13.27% -> 13.65%
		C-OH: 285.9 eV -> 285.8 eV	8.26% -> 7.08%

Table S4 Summary of changes in the XPS spectra of different RGO samples and GO-HM samples before and after Pb adsorption.

Functional group		Peak position (Before to after)	% of total area (Before to after)
COOH	O1s	C=O: 531.2 eV-> 531.2 eV	50.05% ->46.34%
		C-OH: 533.0 eV -> 533.0 eV	49.95% -> 52.69%
		Pb-O: Appears at 530.9 eV.	0% -> 0.97%
	C1s	C=C: 284.4 eV-> 284.4 eV	64.58% -> 69.43%
		C-C: 285.1 eV -> 285.2 eV	8.78% -> 8.50%
		C-OH: 285.8 eV-> 285.9 eV	10.65% ->7.61%
		C=O: 286.6 eV-> 286.7 eV	6.15% ->5.52 %
		O-C=O: 288.4 eV ->288.4 eV	9.84% -> 8.94%
NH	C1s	C=C: 284.4 eV-> 284.4 eV	76.05% -> 73.69%
		C-C/C-N: 285.3 eV -> 285.3 eV	23.95% -> 26.31%
	N1s	C=NH: 398.3 eV -> 398.3 eV	66.12% -> 74.25%
		C-NH_2: 399.8 eV ->399.8 eV	33.88% ->25.75%
OH (OH-RGO)	O1s	C=O: 531.1 eV-> 531.1 eV	32.11% ->44.68%
		C-OH: 533.0 eV -> 533.1 eV	67.89% ->47.97%
		Pb-O: Appears at 530.8 eV.	0% ->7.34%
	C1s	C=C: 284.4 eV-> 284.4 eV	72.78% ->86.26%
		C-C: 285.1 eV -> 285.2 eV	10.23% ->2.29%
		C-OH: 285.6 eV-> 285.6 eV	8.91% ->5.85%
		C=O: 286.2 eV-> 286.3 eV	8.09% ->5.59%
OH (GO-HM)	O1s	C=O: 531.8 eV-> 531.7 eV	39.74% ->22.31%
		C-OH: 532.8 eV -> 532.8 eV	60.26% ->77.69%
	C1s	C=C: 284.4 eV-> 284.4 eV	44.47% ->38.82%
		C-C: 285.5 eV -> 285.5 eV	3.43% ->6.70%
		C-OH: 286.7 eV-> 286.8 eV	38.61% ->37.37%
		O-C=O: 288.3 eV ->288.0 eV	13.50% ->17.11%
None (RGO)	C1s	C=C: 284.4 eV-> 284.4 eV	71.54% ->77.94%
		C=C: 285.2 eV-> 285.3 eV	28.46% ->22.06%

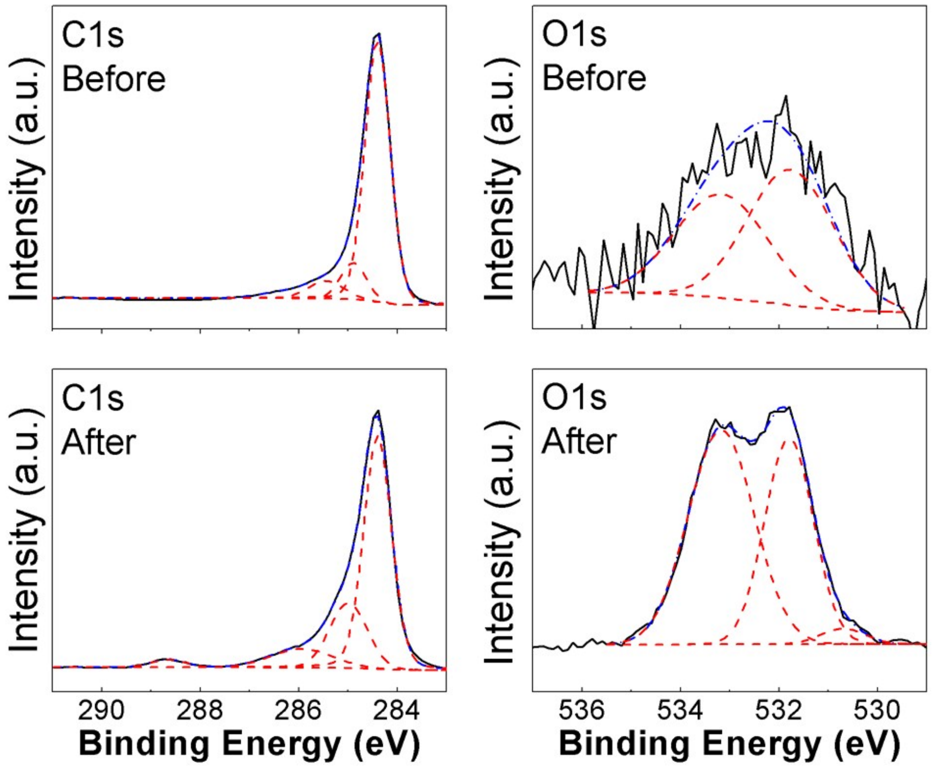

Figure S7 XPS spectra of C1s and O1s peaks of OHCNT samples (1.8%) before and after lead adsorption.

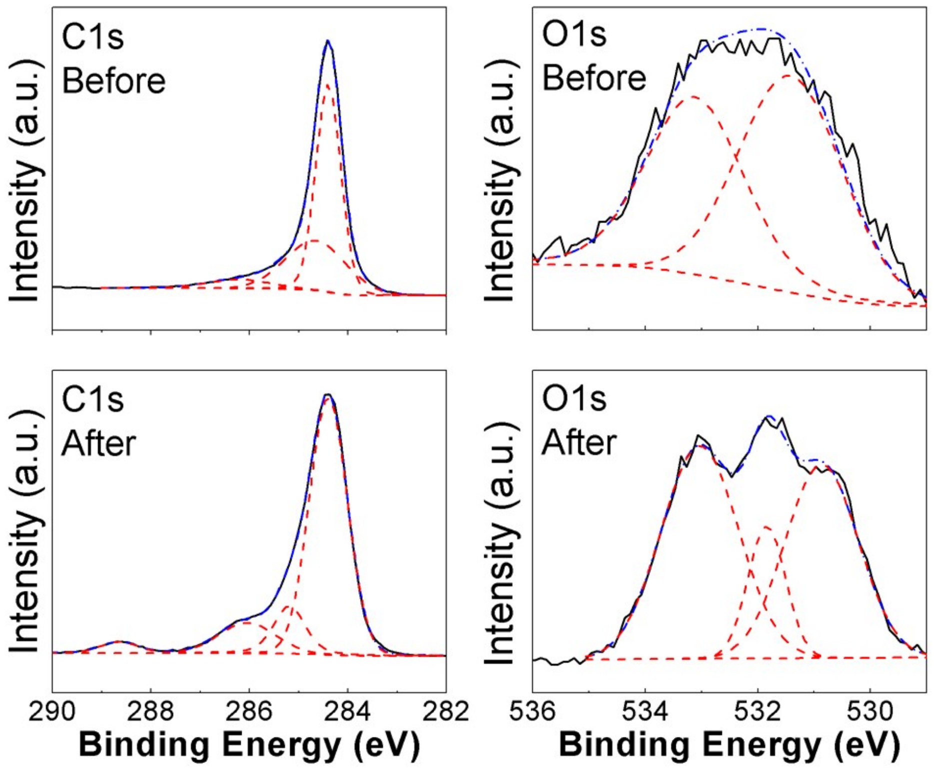

Figure S8 XPS spectra of C1s and O1s peaks of OHCNT samples (5.5%) before and after lead adsorption.

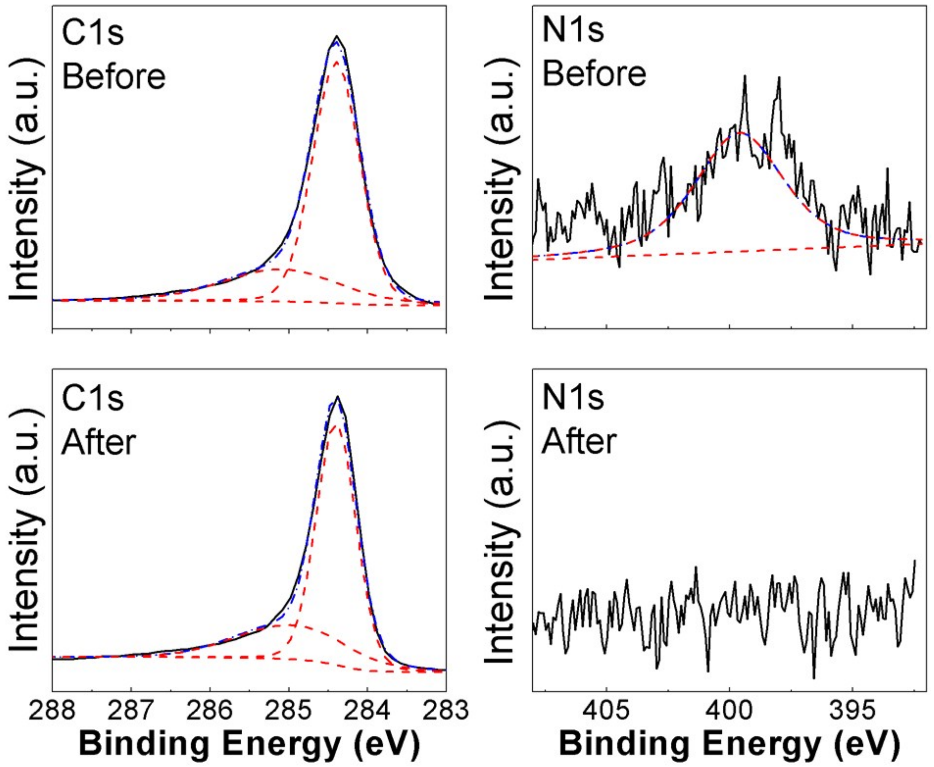

Figure S9 XPS spectra of C1s and N1s peaks of NHCNT samples before and after lead adsorption.

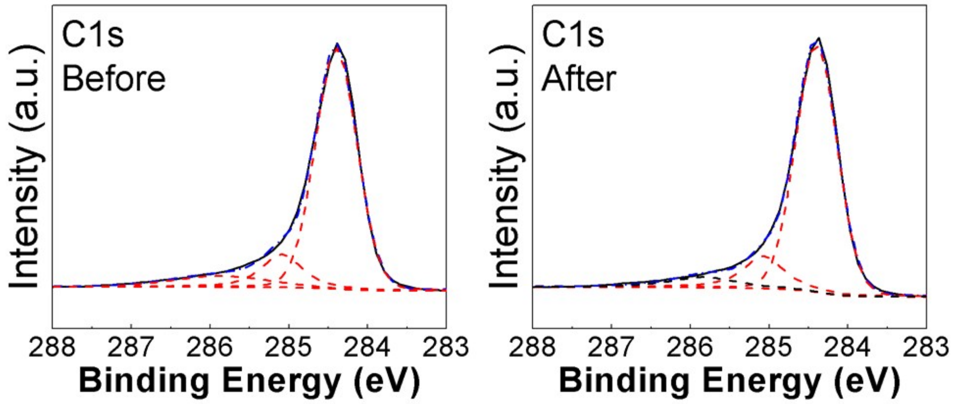

Figure S10 XPS spectra of C1s peak of MWCNT samples before and after lead adsorption.

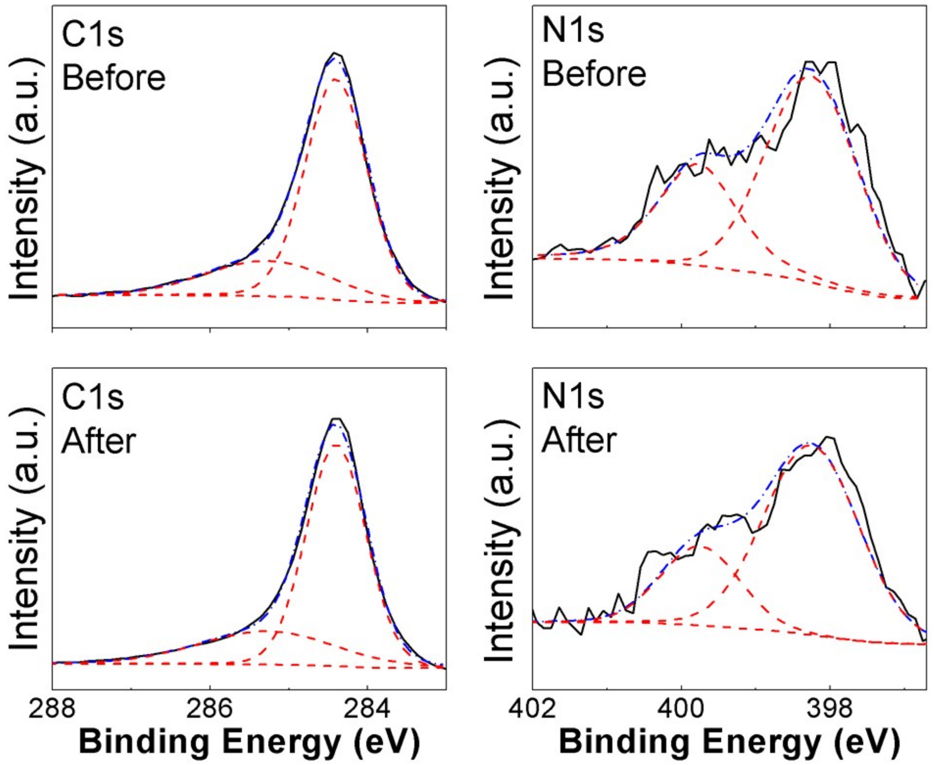

Figure S11 XPS spectra of C1s and N1s peaks peaks of NH-RGO samples before and after lead adsorption.

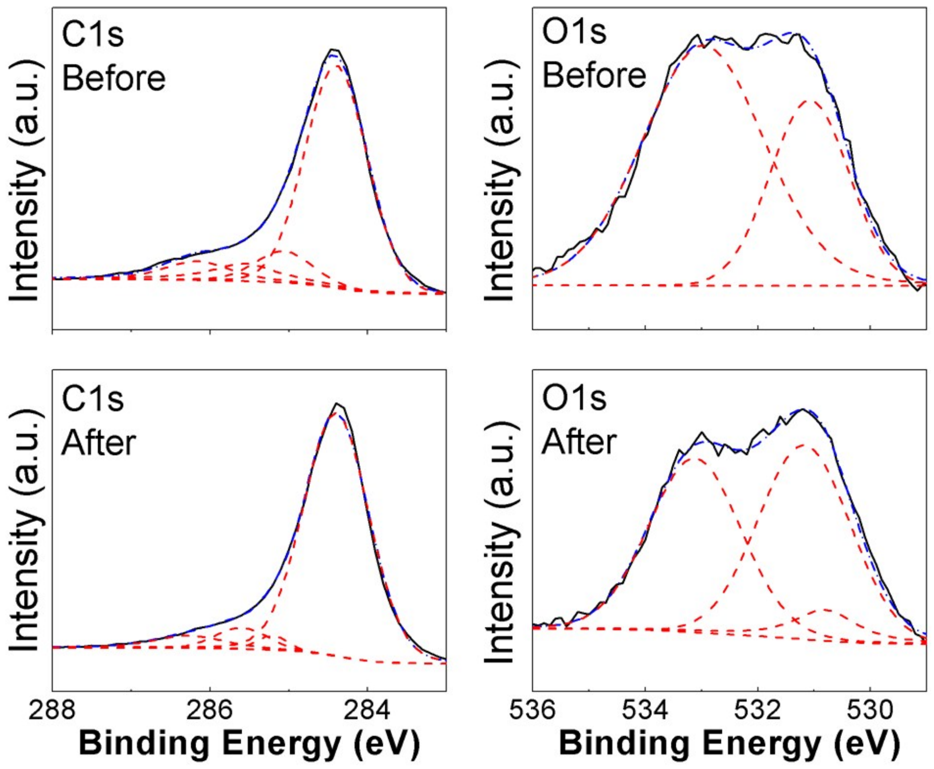

Figure S12 XPS spectra of C1s and O1s peaks of OH-RGO samples before and after lead adsorption.

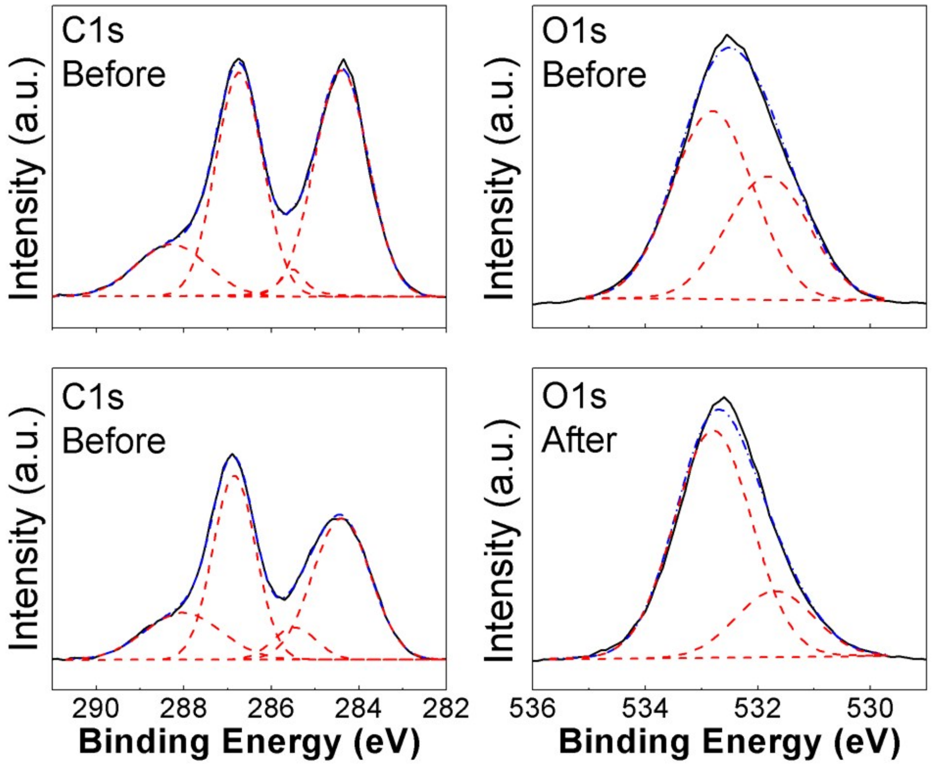

Figure S13 XPS spectra of C1s and O1s peaks of GO-HM samples before and after lead adsorption.

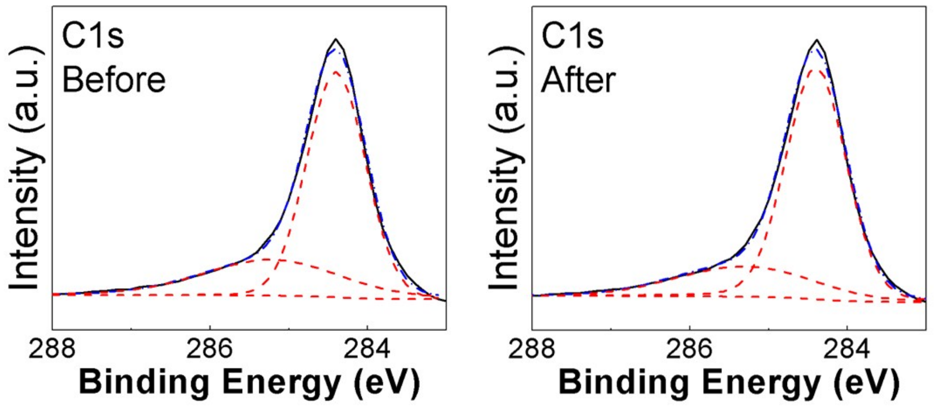

Figure S14 XPS spectra of C1s and O1s peaks of RGO samples before and after lead adsorption.

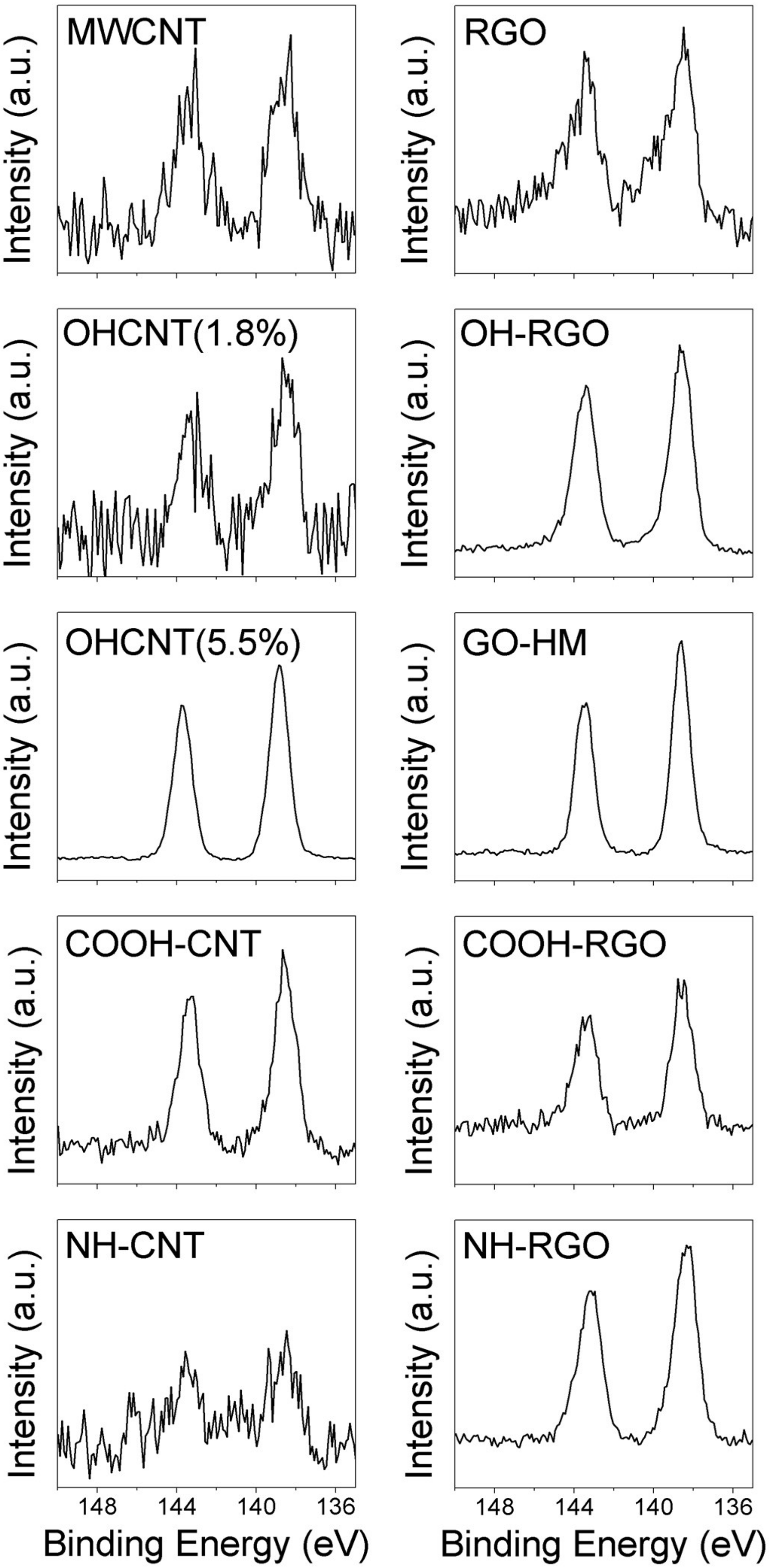

Figure S15 XPS spectra of Pb 4f peak of different nanostructured carbon samples after lead adsorption.
